# Isolation of New 40 Microsatellite Markers in Mandarin Fish (*Siniperca chuatsi*)

**DOI:** 10.3390/ijms12074180

**Published:** 2011-06-24

**Authors:** Xiaolian Liu, Wei Luo, Cong Zeng, Weimin Wang, Zexia Gao

**Affiliations:** Key Lab of Agricultural Animal Genetics, Breeding and Reproduction of Ministry of Education, College of Fisheries, Huazhong Agricultural University, Wuhan 430070, China; E-Mails: xiaolianiss@gmail.com (X.L.); lwdjn@live.cn (W.L.); congzeng@live.cn (C.Z.); wangwm5911@gmail.com (W.W.)

**Keywords:** microsatellite, genomic SSRs, EST-SSRs, *Siniperca chuatsi*

## Abstract

In this study, 23 genomic microsatellite DNA markers and 17 express sequence tag (EST)-derived microsatellites were developed and characterized using the fast isolation by AFLP of sequences containing repeats (FIASCO) method and data mining from public EST databases of mandarin fish (*Siniperca chuatsi*). These polymorphic microsatellite markers were then tested for polymorphism in a wild *S. chuatsi* population. The number of alleles at 23 genomic SSRs varied from 2 to 19 with an average of 8.0 alleles per locus. The average observed and expected heterozygosities were 0.746 and 0.711, respectively. Of 5361 EST sequences examined, 3.9% (209) contain microsatellites, and di-nucleotide repeats are the most abundant (67.0%), followed by tri-nucleotide (29.7%) and tetra-nucleotide repeats (3.3%). The number of alleles at 17 EST-SSRs varied from 2 to 17 with an average of 8.4 alleles per locus. The average observed and expected heterozygosities were 0.789 and 0.685, respectively. No significant difference of loci polymorphism was found between genomic SSRs and EST-SSRs in terms of number of alleles and heterozygosities. Results of cross-species utility indicated that 13 (52.2%) of the genomic-SSRs and 13 (76.5%) of the EST-SSRs were successfully cross-amplified in a related species, the golden mandarin fish (*Siniperca scherzeri*).

## 1. Introduction

Mandarin fish (*Siniperca chuatsi*), belonging to Perciformes sinipercinae, is an endemic freshwater fish species in north-eastern Asian countries. In China, it is mostly distributed in the Yangtze River and the Pearl River [1]. The mandarin fish has been a commercially important and peculiar freshwater fish species in China and is widely cultured throughout the country; it is also important in stocking fisheries in lakes and reservoirs [2]. However, a breeding program aiding to improve its traits such as growth rate and disease resistance has not yet been initiated. Microsatellite marker, also known as simple sequence repeat (SSR), has been considered as one of the efficient molecular markers that provide genetic information due to its co-dominance, high mutation rate, abundance throughout the genome and ease of scoring. Therefore it was widely used in population genetic analysis, genetic mapping and marker-assisted selection of many kinds of fish species [3–5]. Although several genomic microsatellite DNA markers, also known as type II markers, are available for *S. chuatsi* [6,7], the number of microsatellite markers is still insufficient to construct a linkage map for a further study of quantitative trait locus which can aid to marker-assisted breeding of this species. No type I markers which are associated with functional genes, like EST microsatellite markers, have been reported so far in this fish species. In the present study, we developed 23 new polymorphic genomic SSR markers and 17 EST-SSR markers by exploiting EST databases of *S. chuatsi*. Additionally, the cross utility of these markers was tested in a related species, the golden mandarin fish (*Siniperca scherzeri*).

## 2. Results and Discussion

Among 106 positive clones sequenced from the microsatellite-enriched library, 62 clones were found to contain simple sequence repeats, and 38 primer pairs were designed from the sequences with enough flanking region. A total of 5361 *S. chuatsi* EST sequences were obtained from GenBank dbEST and 209 EST sequences containing SSR were derived. Then 32 sequences with enough flanking sequence were selected for primer design. In the wild Poyang Lake population, 23 out of 38 loci isolated from genomic DNA and 17 out of 32 loci developed from EST were polymorphic (Table 1). The average allele number per locus was 8.0 (range from 2 to 19) for genomic SSRs and 8.4 (range from 2 to 17) for EST-SSRs. The average observed and expected heterozygosities were 0.746 (range from 0.000 to 1.000) and 0.711 (range from 0.286 to 0.926) for genomic SSRs, while 0.789 (range from 0.000 to 1.000) and 0.685 (range from 0.064 to 0.923) for EST-SSRs, respectively. The level of polymorphism at these loci was much higher than previously reported microsatellites of *S. chuatsi* in terms of number of alleles [6,7], but when it comes to observed heterozygosity, it was much higher than the results of Kuang *et al*. (0.472) [6], and slightly lower than Zhang *et al*. (0.748) [7]. After sequential Bonferroni correction for multiple tests, five loci showed significant deviation from the Hardy-Weinberg equilibrium (HWE). The presence of null alleles was checked by MICROCHECKER version 2.2.3 software [8], but no evidence for allelic dropout was found in these loci. The heterozygote deficiency at these loci was responsible for the departure of HWE. Another possible explanation for the departure from HWE is the dramatic contemporary decline in spawning populations, and consequent non-random mating and genetic bottlenecks [9]. A final explanation is subpopulation structure which cannot be ruled out without further analysis. No pair of loci was found to be in linkage disequilibrium.

In previous studies, the level of polymorphism of EST-SSRs has usually been observed to be lower than that of genomic SSRs in aquatic species [10,11]. However, in our present study, there is no significant difference of loci polymorphism between EST-SSRs and genomic SSRs in terms of allele number and heterozygosity (*t*-test, *P* > 0.05). Many studies have already demonstrated that the level of polymorphism of SSR often increases with increasing number of repeat units [12,13]. The high polymorphism of EST-SSRs may be correlated with the relatively large number of repeat units (ten units of di- or tri-nucleotide repeat motifs) checked in this study for SSR in *S. chuatsi* EST database.

The first set of EST-SSR for *S. chuatsi* is described in this study. Of 5361 ESTs of *S. chuatsi* downloaded from GenBank database, 209 (3.9%) sequences contain a microsatellite locus. Analysis of the repeat motif showed that di-nucleotide repeats were the most abundant (67%), which was slightly lower than some fish species such as yellow perch *Perca flavescens* [10] and large yellow croaker *Pseudosciaena crocea* [14], while much higher than bay scallop *Argopecten irradians* [15] and common carp *Cyprinus carpio* [16]. Tri-nucleotide and tetra-nucleotide repeats accounted for 29.7% and 3.3% of the total microsatellites, respectively. Among di-nucleotide repeats, (AC)*_n_* repeats were the most abundant, followed by (AG)*_n_* repeats and (TA)*_n_* repeats, with the same findings in large yellow croaker [14].

Cross-species amplification was conducted in a related species *S. scherzeri*. Of the 17 EST-SSRs and 23 genomic SSRs primers tested, 13 (76.5%) and 13 (52.2%) were successfully amplified, respectively. The number of alleles per locus checked in 10 individuals ranged from 1 to 6 in *S. scherzeri*. The rate of successful cross-amplification in EST-SSRs was higher than genomic SSRs, indicating that EST-SSRs had higher amplification transferability than genomic SSR markers [10,16].

## 3. Experimental Section

### 3.1. EST Database Mining

*S. chuatsi* EST sequences were obtained from GenBank dbEST (http://www.ncbi.nlm.nih.gov/nucest?term=EST). The SSR sequences were screened using the SSRHunter 1.3 program (http://www.bio-soft.net/dna/) and the criteria for SSRs were set as sequences having at least ten units of dinucleotide repeat motifs, five units of tri- and tetra-nucleotide repeat motifs, and four of hexanucleotide repeat motifs. Then the primers were designed for EST-SSR locus with ten units of di- or tri-nucleotide repeat motifs using software PRIMER 3 [17].

### 3.2. Isolation of Genomic Microsatellites

Total genomic DNA was extracted from tail fin using a traditional proteinase-K digestion and phenol-chloroform protocol with RNase treatment [18]. Subsequently, a partial genomic library enriched for AC-microsatellite was constructed using fast isolation by AFLP of sequences containing repeats (FIASCO) method [19]. In brief, approximately 200 ng of total genomic DNA was digested with *Mse*I enzyme (New England BioLabs). The fragments within the size range of 200–1000 bp were recovered from an agarose gel and ligated to *Mse*I AFLP adaptors (5′-GACGATGAGTCCTGAG-3′/5′-TACTCAGGACTCAT-3′) using T4 DNA ligase (New England BioLabs). The digestion-ligation mixture was diluted (1:10) and directly amplified using *Mse*I-N primers (5′-GATGAGTCCTGAGTAAN-3′) to give numerous copies of each fragment. PCR was performed in a 20 μL reaction mixture including 100 ng DNA, 1 × PCR buffer, 1.5 mM MgCl_2_, 200 μM dNTPs, 0.5 μM of each primer, 1 U Taq polymerase (TIANGEN), and by denaturing at 94 °C for 4 min, followed by 30 cycles of 1 min at 94 °C, 30 s at 50 °C, 1 min at 72 °C and an extension at 72 °C for 7 min. After denaturation at 95 °C for 10 min, PCR products were hybridized to 5′-biotinylated probe (AC)_8_ or (CT)_8_ oligonucleotide probe in hybridization solution with 6 × saline-sodium citrate (SSC) and 0.1% SDS (sodium dodecyl sulfate) at 55 °C for 30 min. Probe-bound DNA fragments were then enriched by Streptavidin MagneSphere Paramagnetic Particles (Promega) at room temperature for 30 min, followed by several non-stringent (10 mM Tris-HCl, 1 mM EDTA, 1 mM NaCl) and stringent washes (SSC 0.2× and 0.1% SDS) to remove non-specifically binding and unbound DNA. Captured DNA fragments were amplified with *Mse*I primer and the same cycling program as pre-hybridization PCR. The PCR products were ligated to pMD18-T plasmid vector (TaKaRa) and transformed into competent *Escherichia coli* DH5a cells (Invitrogen). Positive clones were screened by blue/white selection and tested by PCR amplification using *Mse*I primer and M13 universal primer. In total, 106 positive clones were sequenced with T7 primer in one direction using ABI Prism 3730 automated DNA sequencer (Applied Biosystems).

### 3.3. PCR Amplification and Genotyping

All sequences containing simple sequence repeats and enough flanking sequence, including genomic and EST sequences were selected for primer design by using software PRIMER 3. PCR conditions were optimized for each pair of primers. Then polymorphism of microsatellite loci was evaluated on 33 wild samples collected from the Poyang Lake (28°22′–29°45′ N and 115°47′–116°45′ E) in Jiangxi province, China. The PCR were carried out in 20 μL volumes containing 1 × Taq buffer, 1.5 mM MgCl_2_, 200 μM dNTPs, 0.5 μM of each primer, 1 U Taq polymerase, 200 ng genomic DNA. PCR thermal conditions were as follows: an initial denaturation step of 5 min at 94 °C, followed by 35 cycles of 30 s at 94 °C, 30 s at locus-specific annealing temperature (see Table 1), 45 s at 72 °C, followed by a final extension at 72 °C for 5 min. PCR products were separated on a 8% non-denaturing polyacrylamide gel electrophoresis and visualized by silver staining. A 25 bp DNA ladder (Promega) was used to identify alleles.

### 3.4. Data Analysis

Number of alleles (*N*_a_), expected (*H_E_*) and observed heterozygosities (*H*_o_), were calculated using POPGENE 32 software [20]. Deviations from Hardy–Weinberg equilibrium (HWE) for each locus, linkage disequilibrium (LD) between all loci were tested by online version GENEPOP (http://genepop.curtin.edu.au/) [21]. All results were adjusted for multiple simultaneous comparisons using a sequential Bonferroni correction [22].

## 4. Conclusions

In summary, 40 polymorphic microsatellite markers, including EST-SSR and genomic SSR were developed in this study. Particularly, mining the EST database provides an efficient and time-saving approach to obtain new microsatellite markers for species of interest. In further studies, these markers would be useful for population genetics, parentage analysis, association analysis, functional genes cloning and the construction of a linkage map of *S. chuatsi* and its related species.

## Figures and Tables

**Table 1:** Characterization of 40 microsatellite loci in 33 *Siniperca chuatsi* individuals.

Loci	GenBank Accession No.	Primer Sequence (5′-3′)	Repeat Motif	*T*_a_	*S* (bp)	*N*_a_	*H*_o_/*H*_E_	*N*_a_ in *Siniperca scherzeri*
EST-1	GR477481	F: CCAGCCAACAACCATAAAG	(CA)_10_	58	200–240	6	0.648/0.632	2
		R: CCCAGGTAGAAGACCGTGA						
EST-4	GR477363	F: GTCAGTCCATCAGCCATTA	(CA)_19_	53	263–370	13	0.970/0.834	2
		R: TTCCGATGAAGAGTCACCAC						
EST-5	GR477357	F: TTGCTGCGTTAAAGGGTT	(TC)_26_	50	319–353	2	0.000 */0.064	2
		R: CTTGTGGTCGAATGTGCC						
EST-6	GR477337	F: TCCCAGTAGCATTCAAAC	(TG)_11_(GT)_6_	61	135–198	8	0.754/0.765	6
		R: TGCATACATACACCCACA						
EST-7	GR477325	F: AAAGCAACGCAGTGTCTC	(AC)_11_	61	178–212	7	0.783/0.774	2
		R: GCAATCCCTTCTTCTTCTC						
EST-10	GR477249	F: GGCTGTTCTTGTTCCCTG	(GT)_11_(GT)_5_(GT)_6_	55	201–331	17	0.898/0.867	4
		R: CCCAAATACATGCCCTCA						
EST-17	GR476891	F: GAAGCCGGAGTGGACTGT	(CT)_33_	61	288–341	8	0.857/0.761	2
		R: GGTTTCGGGTTGAGGAGA						
EST-19	GR476867	F: GACAGTACAAGTAAGGCACA	(CT)_7_(CT)_11_(TG)_14_	61	285–336	7	0.697/0.766	1
		R: GTCGCATAAATATCACAGAA						
EST-21	GR476843	F: AGTGAGGTGGAGGGGTGA	(CA)_15_	63	128–230	17	1.000/0.923	–
		R: TACGTTGCCGATGAAAGC						
EST-24	GR476700	F: TGCCAATAAGGGTTTCTA	(TG)_5_(TG)_11_	55	208–332	15	1.000/0.905	3
		R: GACACTCTTTCGCTCTGC						
EST-26	GR476421	F: CATCATGGCAGCATCAGT	(TC)_26_	63	156–186	2	0.511/0.484	–
		R: GCGAGGTAACCCAGGAGA						
EST-31	GR476370	F: AGCATCATAGGCCAGCAC	(AC)_19_	50	272–276	2	0.000*/0.437	–
		R: CCGCCATATTAGGTTCTC						
EST-33	GR476286	F: CACTGTGCTCAACGTACT	(AC)_13_	63	126–144	4	0.528/0.531	1
		R: GTGACATTTAGCCCATAA						
EST-35	GR476157	F: CCATAGTTTGTGGTGGTA	(TA)_21_	55	208–256	10	0.858/0.852	–
		R: CTGGAGGAAATAAAGGAG						
EST-42	GR476074	F: ATTTGGCTATTCACTCTTC	(GT)_10_(AG)_17_(GA)_5_(AG)_12_(TTA)_6_	58	152–234	8	0.684/0.769	4
		R: CTCTTTCTCGCTCTGTCT						
EST-43	GR476056	F: AAAGTCCCTGATACATAG	(TA)_18_	55	184–306	14	0.860/0.856	2
		R: GTATTCATGGGTTTGGTT						
EST-57	GR478790	F: CAGCAGCATCACCTTCACC	(ACA)_10_	50	174–200	2	0.000 */0.422	1
		R: GCTGGGCTTTGAGGGTAGA						
Mar1	GU324507	F:TCAGTGGAAAATAATGAAAAGGAAG	(CA)_5_	58	162–238	6	0.757/0.754	2
		R:TAGTGAGTGTGGGTGTAGGTGGGTT						
Mar3	GU324508	F:GTAACCCACCTACACCCACACTCAC	(CA)_17_	58	145–194	4	0.741/0.746	–
		R:CACTGTCACAGGAATGAAGAAAACT						
Mar5	GU324510	F:ATAGACTTTACACACATCACTGGAG	(AC)_5_(AC)_10_	55	279–385	19	0.922/0.926	2
		R:AGAGAGAAGAGAGTGTGATGAAGAG						
Mar9	GU324513	F:GACATCACCAATACCTCCTGACACG	(GT)_21_	52	232-397	19	1.000/0.899	–
		R:TACACACGCATGGAGTATCTGGATC						
Mar10	GU324514	F:GATTGGCACCTTGAAACACGCATAC	(GT)_19_	52	84–160	16	0.895/0.888	5
		R:GTGAACATAACTCATTTCTGCCAGG						
Mar12	GU324516	F:AGATAACACGATAGATTGATACACG	(GT)_12_	55	175–233	14	0.871/0.862	2
		R:CACTTGATGACCTGTCCTTACTATG						
Mar13	GU324517	F: CAGGCACAGACTCACACACT	(TG)_6_	52	163–240	11	0.892/0.886	2
		R: GTGCCTGTCTGAGCGTGA						
Mar14	GU324518	F: CACACACTCTCGCACACTCG	(TG)_5_	52	65–173	2	0.462/0.500	1
		R: ACGGTACGCACCTCTGTCAC						
Mar8	GU324512	F:TGCGATAACAGGATACCAGTAATGC	(CA)_7_	50	154–201	5	0.611/0.544	2
		R:CAGAGGTCAAACGGGTGAAGAGG						
Mar11	GU324515	F:TTTGCTACCACAACAGGAAGAACAT	(GT)_18_	61	138–170	6	0.739/0.743	–
		R:TTTGAAAAATGATTAAGGGAGACAT						
Mar4	GU324509	F:TAGGGTAAGGGACTAGGGAACGCAT	(GT)_6_(TG)_10_(GA)_16_(AG)_5_	52	230–308	7	0.764/0.714	–
		R:TACACAAGCCCAAAGATTCTCAAGC						
Mar6	GU324511	F: TACCTTCGCTTCTCATGTGC	(TG)_14_	53	110–158	2	0.346*/0.286	–
		R: TGAGTGGTGCATTGTGTGTG						
GY09	HQ875477	F: CGCTACCACTTCCCACA	(CT)_13_(CA)_22_	53	354–402	3	0.587/0.610	–
		R: TAGACTCCCATTTCCACCA						
GY12	HQ875479	F: AAGTAGCAGAAAATGGAAAT	(TC)_10_	50	316–400	10	0.802/0.805	2
		R: CTCAGGTGGAACAATCATC						
GY13	HQ875480	F: TGTTAGACCTGCTGGAGT	(AC)_16_(GA)_12_	61	200–327	12	0.833/0.851	2
		R: GAGGAGGCTGTAAATCG						
GY14	HQ875481	F: ATCAGTGGGCAGGGT	(AG)_12_(AG)_5_	55	272–382	6	0.646/0.651	–
		R: AAAGCGAGCGCTAGA						
GY15	HQ875482	F:AGTCGTATGGGTTGGTG	(AC)_6_AGT(CA)_9_CT(CA)_8_GT (CA)_7_CG(CA)_18_	50	264–348	2	0.466/0.500	1
		R: ATGGTATCAGTCAGGGTG						
GY16	HQ875483	F: TTAAGCAGCGTTACCTAAT	(TG)_5_	50	164–200	4	0.645/0.650	2
		R: AGACGGGAATCTGTGAA						
GY17	HQ875484	F: CGATGTCCACTCGAACT	(GT)_79_(GC)_7_(CA)_5_	50	170-239	8	0.742/0.735	–
		R: CCTCATCTTCAGCCACG						
GY18	HQ875486	F: GATAGAGGCAGAAACACC	(TG)_17_	50	116-166	4	0.571/0.573	1
		R: ATACGGCATATTGGAAA						
GY19	HQ875487	F: ACTGGTAACCACAGAACAT	(AC)_15_	63	180-247	10	0.845/0.846	–
		R: ATTGCTTAATTGGACACTC						
GY20	HQ875488	F: TTGGTGTATTGAAGCA	(GT)_30_	50	138-202	11	0.870/0.863	2
		R: AAAGCCAGCAGCA						
GY22	HQ875490	F: CAGTGAATGGACAGTTTGGT	(AC)_10_	55	200-225	3	0.000 */0.523	–
		R: CTCTGCATGTTTGATTGAGG						

*T*_a_, annealing temperature (°C); *S*, allele size range; *N*_a_, number of alleles; *H*_O_, observed heterozygosity; *H*_E_, expected heterozygosity;

* indicated deviation from Hardy-Weinberg equilibrium (*P* < 0.05) after Bonferroni correction.
